# CdTe Quantum Dots Modified with Cysteamine: A New Efficient Nanosensor for the Determination of Folic Acid

**DOI:** 10.3390/s19204548

**Published:** 2019-10-19

**Authors:** Doris E. Ramírez-Herrera, Ana Patricia Reyes-Cruzaley, Giselle Dominguez, Francisco Paraguay-Delgado, Antonio Tirado-Guízar, Georgina Pina-Luis

**Affiliations:** 1 Tecnológico Nacional de México/Instituto Tecnológico de Tijuana, Centro de Graduados e Investigación, A.P. 1166, Tijuana 22500, Mexico; doris_e_777@hotmail.com (D.E.R.-H.); ana.reyes@tectijuana.edu.mx (A.P.R.-C.); guizarantonio@gmail.com (A.T.-G.); 2 Miami Dade College, North Campus, 11380 NW 27th Ave, Miami, FL 33167, USA; gdomingu@mdc.edu; 3 Centro de Investigación en Materiales Avanzados S. C., Departamento de Física de Materiales, Av. Miguel de Cervantes 120, Complejo Industrial Chihuahua, Chihuahua CP 31136, Mexico; francisco.paraguay@cimav.edu.mx

**Keywords:** folic acid nanosensor, positive quantum dots, fluorescence quenching, electron transfer

## Abstract

In this paper, we report the synthesis, characterization, and application of a new fluorescent nanosensor based on water-soluble CdTe quantum dots (QDs) coated with cysteamine (CA) for the determination of folic acid (FA). CdTe/CA QDs were characterized by high-resolution transmission electron microscopy, the zeta potential, and Fourier-transform infrared (FT-IR), UV-visible, and fluorescence spectroscopy. CdTe QDs coated with mercaptopropionic acid (MPA) and glutathione (GSH) were prepared for comparison purposes. The effect of FA on the photoluminescence intensity of the three thiol-capped QDs at pH 8 was studied. Only CdTe/CA QDs showed a notable fluorescence quenching in the presence of FA. Then, a nanosensor based on the fluorescence quenching of the CdTe QDs at pH 8 was explored. Under optimum conditions, the calibration curve showed a linear fluorescence quenching response in a concentration range of FA from 0.16 to 16.4 μM (R^2^ = 0.9944), with a detection limit of 0.048 μM. A probable mechanism of fluorescence quenching was proposed. The nanosensor showed good selectivity over other possible interferences. This method has been applied for FA quantification in orange beverage samples with excellent results (recoveries from 98.3 to 103.9%). The good selectivity, sensitivity, low cost, and rapidity make CdTe /CA QDs a suitable nanosensor for FA determination.

## 1. Introduction

**Figure 1:**
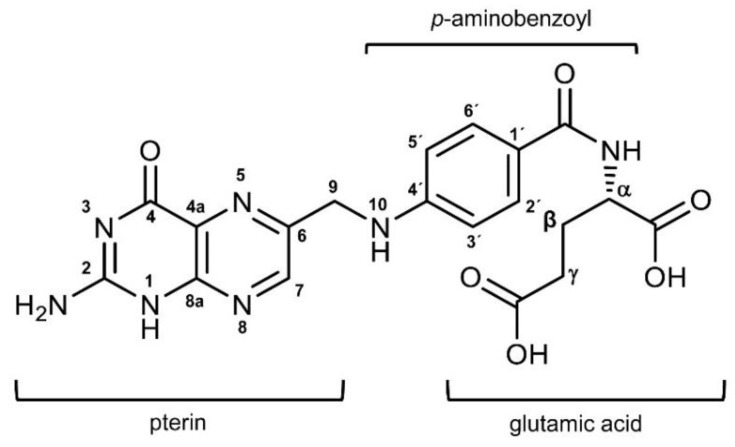
Structure of folic acid.

Folic acid (FA) is a known water-soluble B-vitamin comprising three sub-components: A pterin ring, p-aminobenzoyl residue, and L-glutamic acid (Figure 1) [1]. It is linked with different diseases; FA prevents birth defects of the brain and spinal cord, especially spina bifida, and decreases serum homocysteine levels, reducing the risk of heart diseases [2,3]. FA is an essential vitamin that is nowadays prescribed to pregnant women and is indicated in the prevention of certain types of anemia [4,5]. FA is also used in bioimaging as an element of orientation toward cancer cells, as folate receptors are highly over-expressed on the surface of many tumors [6,7]. FA plays an important role in the proper development of physiological processes in the body [8]. 

The human body is not able to synthesize FA, and its presence in foods such as spinach, cereals, citrus, rice, and beans may not be enough to meet the daily requirement for this vitamin [3]. The daily consumption of foods fortified with FA, such as citrus beverages, can provide the amount required for human health. Therefore, having a simple and fast method for determining FA in commercial beverages is of great interest.

Different methods have been reported in the literature for the quantification of FA, including spectrophotometric methods [9,10], electrochemical techniques [11,12], high-performance liquid chromatography (HPLC) [13,14,15] and ultrahigh HPLC, and ion chromatography [16]. In general, these methods are based on time-consuming reactions that involve laborious and slow procedures. In particular, the chromatographic methods require expensive equipment and the use of large volumes of organic solvents. Therefore, the development of a simple, accurate, and low-cost method with high sensitivity and selectivity for the determination of FA is of great importance for monitoring the quality of food products. Fluorescence methods have gained considerable attention in recent years, due to their selectivity, sensitivity, easy sample preparation, and rapid analysis. As a kind of new fluorophore, quantum dots (QDs) are a favorable alternative as they have advantages over conventional organic fluorescent dyes. They have unusual optical and electronic properties as a consequence of the quantum confinement effect. QDs have been proven to be effective in different applications as biological biomarkers, sensors, drug delivery systems, and solar cells [17,18,19,20,21].

In recent years, methods for the determination of FA based on the use of nanomaterials have been reported. Graphene oxide/Ag nanoparticle hybrids with surface-enhanced Raman spectroscopy (SERS) detection [22], a bovine serum albumin (BSA)-modified gold nanocluster with fluorescence detection [23], and a ZrO_2_ nanoparticle-modified carbon paste electrode with voltammetry detection [24] have been used in the determination of FA. Other works show the determination of FA using QDs capped with thiolated ligands as thioglycolic (TGA) [25] and mercaptopropionic (MPA) acids [26]. However, in these nanoparticles, at pH 8, the carboxylate groups are dissociated, giving a negative charge to the surface of the QDs. Under these conditions, FA is also negatively charged, and the interaction between the QDs and FA is not favored, due to electrostatic repulsions. Therefore, QDs coated with amino groups, which, at pH 8, are positively charged, could improve the analytical characteristics of the sensor. 

Cysteamine (CA) is a bifunctional ligand, with thiol groups that bind strongly to metal ions on the surface of QDs and hydrophilic amino groups that render the QDs biocompatible and dispersible in water [27]. In addition, the amino groups of CA are protonated at physiological pH and can interact with negatively charged molecules, favoring the formation of a stable assembly by electrostatic interactions. 

There are some works based on amino-coated QDs as nanosensors for the determination of different molecules [27,28,29,30]. However, to our knowledge, there are no reports based on CdTe/CA QDs for the determination of FA. Therefore, the objective of the present work is the development of a sensor based on QDs coated with amino groups and its application in the determination of FA in commercial beverages. The proposed design should improve the analytical characteristics of the sensor. 

**Scheme 1:**
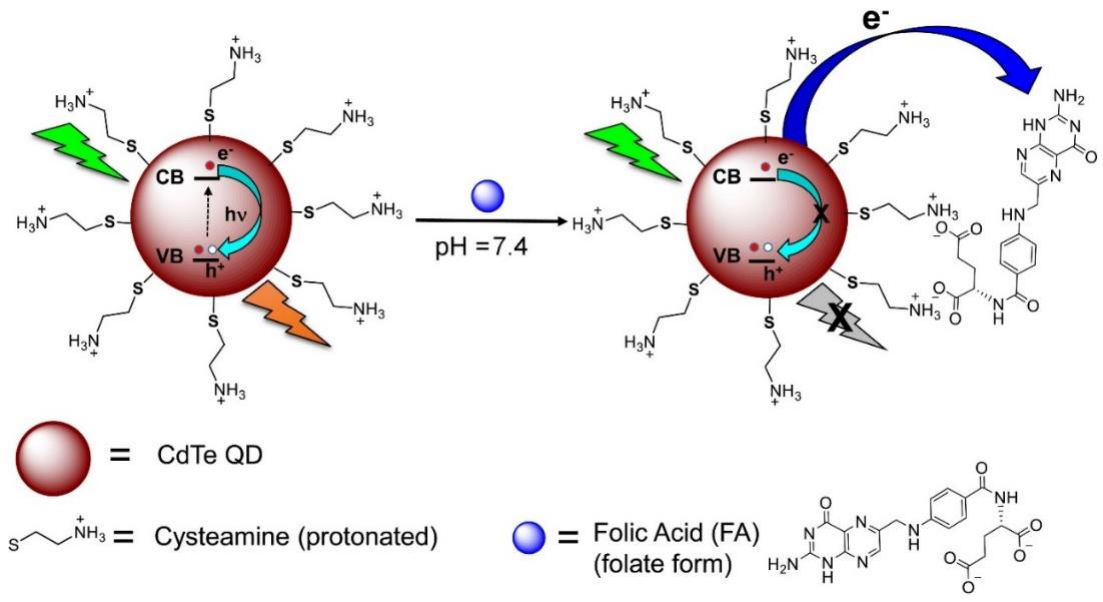
Schematic illustration of quantum dots (QDs)/cysteamine (CA) quenching by folic acid (FA).

In this work, we present a system based on water-soluble CdTe QDs coated with CA for the determination of FA. CdTe/CA was prepared and characterized by high-resolution transmission electron microscopy, FT-IR spectroscopy, the zeta potential, and UV-visible and fluorescence spectroscopy. The analytical response of CdTe QDs capped with CA toward FA was studied and compared to the results obtained using other thiol-capped QDs. As CdTe/CA QDs showed the largest quenching efficiency, this system was further studied for FA determination. The principle of this nanosensor is illustrated in Scheme 1. The probe is based on the fluorescence quenching of CdTe/CA QDs by FA at pH 8. A probable mechanism of fluorescence quenching based on the coordinated behavior of electrostatic interactions between positively charged CdTe/CA QDs (ethylamine groups, pKa = 10.52) and negatively charged FA (carboxylic groups, pKa_α_ = 3.46 and pKa_γ_ = 4.83) and the electronic transfer of QDs to FA is proposed. The nanosensor is rapid, easy to obtain, and shows a low detection limit, high selectivity, and low cost. The method showed excellent results in detecting FA contents in beverages (recoveries from 98.3 to 103.9%). The system provides a new approach for the construction of nanosensors with high application potential in different areas.

## 2. Materials and Methods

### 2.1. Reagents

Cadmium chloride hemi(pentahydrate), potassium tellurite, cysteamine (CA), mercaptopropionic acid (MPA), glutathione (GSH), sodium borohydride (NaBH_4_), sodium hydroxide, ascorbic acid, citric acid, glutamic acid, tartaric acid, glucose, bovine serum albumin (BSA), and fluorescein (quantum yield (QY), 79%) were purchased from Sigma-Aldrich, Mexico. All chemicals (analytical grade) and solvents (spectroscopic grade) were used without further purification. Deionized water was used in the experiments. Beverage samples were obtained from a local supermarket.

### 2.2. Characterization of Thiol-Capped Nanoparticles

Fourier-transform infrared (FT-IR) spectra (650–4000 cm^−1^) were recorded in the transmission mode on a Perkin Elmer FT-IR Spectrum 400 spectrophotometer (Perkin Elmer, Mexico). UV-visible spectra were collected on a Shimadzu spectrophotometer model UV 2700 (Shimadzu, Japan) using a 1 cm path-length quartz cell. Fluorescence spectra were recorded from 300 to 700 nm on a Horiba Nanolog fluorescence spectrophotometer (HORIBA Scientific, Edison, NJ, USA) using a xenon lamp as the excitation source and a 1 cm path-length fluorescence quartz cell. The morphologies and sizes of nanoparticles were characterized by JEM-2200FS (JEOL, Akishima, Japan) transmission electron microscopy (TEM). The microscope was capable of spherical aberration correction in a scanning transmission electron microscope (STEM) mode working at an accelerating voltage of 200 keV. The images were acquired by a high-angle annular dark-field (HAADF) detector. Zeta potential measurements were acquired using a Horiba Scientific SZ-100 nanoparticle analyzer (Horiba Scientific, Japan). A Thermo Scientific pH meter was utilized to measure the pH of the solutions (Thermo Fisher Scientific, Mexico City, Mexico).

### 2.3. Preparation of GSH, MPA, and CA-Capped CdTe QDs

GSH, MPA, and CA-capped CdTe nanocrystals were synthesized according to the methodology used in previous work from the group [31]. Experimental details for the preparation and characterization by UV-visible and fluorescence spectroscopy of QDs modified with MPA, GSH, and CA are described in the Supplementary Material. 

The relative quantum yield (QY) of thiol-capped CdTe QDs was examined by a procedure described in the Supplementary Material.

### 2.4. Stability Study

The photochemistry stability was investigated by exposing the nanomaterial to UV irradiation (365 nm) at a constant temperature with a xenon lamp of 450 W. The dispersed material was prepared in ultrapure water at pH 8 using a phosphate buffer (PBS). The fluorescence intensity was measured using the “kinetic” mode of the Nanolog spectrofluorometer for 60 min.

### 2.5. Fluorescence Study

The fluorescent response of CdTe/CA QDs toward FA was studied by adding 2 mL of QD solution and 1 mL of PBS buffer solution (pH 8) in a 1 cm optical-path quartz fluorescence cell. The titration was carried out by adding increasing amounts of FA in a concentration range from 0 to 16.4 µM. The fluorescence spectra were obtained in a range of emission wavelengths from 500 to 750 nm, at an excitation wavelength of 440 nm. 

### 2.6. Interference Study

The response of the nanoprobe to other compounds was studied through fluorescence spectra. Competition assays were performed for BSA, GSH, glucose, ascorbic, and citric acids. For competition experiments, the solution of CdTe/CA QDs (3 mL) was placed in a 1 cm optical-path quartz cell and mixed with FA solution in the presence of the possible interference, at a molar ratio of FA:Interference of 1:20. The fluorescence intensity was measured at λ_em_ = 597, with excitation at 440 nm.

### 2.7. Sensitivity Detection

For the construction of the calibration graph, FA stock solution was prepared in phosphate buffer (100 µM, pH 8). QD/CA solution, prepared as shown above, was mixed with different concentrations of FA from 0.16 to 16.4 µM in 5 mL volumetric flasks and diluted to the mark. The calibration curve was the average of three repetitions.

### 2.8. Detection of FA in Real Samples

The determination of FA was carried out directly in samples of orange beverages without any previous treatment. The QD solution was mixed with 100 µL of the sample in a buffer solution (pH 8). A standard addition method was used for FA determination. The standard addition curve was obtained in a concentration range between 0 and 7 µM and was the result of three repetitions (R^2^ = 0.9950 sample 1 and R^2^ = 0.9994 sample 2).

## 3. Results and Discussion

### 3.1. Spectral Characteristics of CdTe QDs

The CdTe QDs coated with MPA and GSH were obtained for comparison purposes. CA-coated QDs were obtained to be studied as a FA detection probe. CA was selected as the coating agent because this ligand is bifunctional; it has thiol groups that bind with metal ions on the surface of QDs and also has hydrophilic amine groups to increase the dispersibility of QDs in water [27]. In addition, the amino groups of CA, at physiological pH, are protonated, and the FA is negatively charged, favoring the formation of a stable assembly. 

The typical evolution of the UV-visible absorption and fluorescence spectra of thiol-capped CdTe QDs at different reaction times is shown in Figure S1. Table S1 shows the optical properties of CdTe QDs coated with MPA, GSH, and CA. As can be seen, the absorption and emission spectra of the QDs were red-shifted as the reaction time increased. In addition, narrow and symmetrical emission bands were observed, indicating monodisperse and homogenous QDs. As is known, the reaction time has a significant influence on the size and quantum yield (QY) of the QDs [32]. As the reaction time increases, QDs with a larger size and higher QY are obtained, reaching the maximum values at a given reaction time. The highest QY was observed for the CdTe/CA QDs obtained at a reflux time of 60 min, corresponding to QDs of 3.2 nm in size. Then, these QDs were selected for further studies. This is an important aspect to consider, as QDs with high QY should contribute to the detection efficiency.

**Figure 2:**
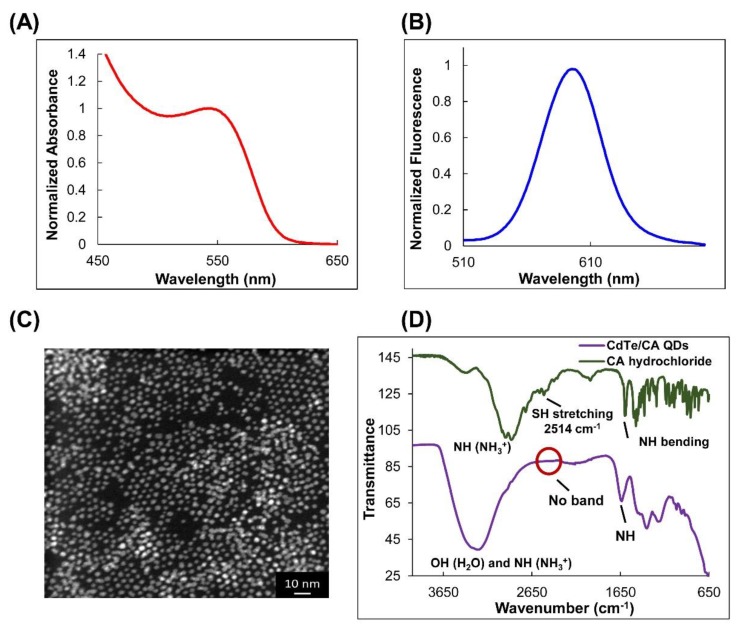
(**A**) Absorption and (**B**) fluorescence emission spectra, (**C**) high-angle annular dark-field (HAADF) image from CdTe/CA QDs, and (**D**) FT-IR spectra of CA hydrochloride (green) and CTe/CA QDs (purple).

Figure 2A,B show the UV-visible absorption and fluorescence spectra of CdTe/CA QDs obtained at a reaction time of 60 min. The absorption spectrum shows an excitonic peak at 545 nm and a strong emission band at 597 nm. The emission band shows a full-width at half-maximum (FWHM) of about 50 nm. The absorption band at 545 nm is assigned to the 1S(h) → 1S(e) in CdTe QDs, which is due to the transition of an electron from the valence band (VB) to the conduction band (CB). 

The diameters of the CdTe QDs obtained were estimated using the empirical Peng regression [33]. The equation for CdTe QDs is shown below:

*D* = (9.8127 × 10^−7^) λ^3^ − (1.7147 × 10^−3^) λ^2^ + (1.0064) λ − 194.84, (1)

where *D* (nm) is the particle size of QDs and λ (nm) is the wavelength of the first excitonic absorption peak of the corresponding QD. The molar absorptivity *ε* was determined using Equation (2) [33].

*ε* = 10043 (*D*)^2.12^. (2)

The results show that the nanoparticles have a diameter of 3.2 nm, corresponding to the emission maximum at 597 nm. The STEM image of as-prepared CdTe/CA QDs (Figure 2C) indicates uniformly dispersed spherical QDs with an average diameter around 3.3 nm, confirming the results obtained by Peng regression.

To confirm the incorporation of CA on the surface of the QDs, FT-IR spectra of CA hydrochloride and CdTe/CA QDs were obtained. As can be seen in Figure 2D, the CA spectrum shows a characteristic NH bending band at 1593 cm^−1^, SH stretching at 2550 cm^−1^, bands of CH_2_ stretching (2950–2750 cm^−1^) and bending (1470 cm^−1^), and CN stretching at 1243 cm^−1^. The absorption between 3300 and 2800 cm^−1^ corresponds to the stretching (asymmetric and symmetric) in the NH_3_^+^ group of the CA hydrochloride. There are similarities between both spectra, but the band at 2550 cm^−1^ corresponding to SH stretching is not present for the CdTe/CA QDs, indicating the covalent incorporation of the CA on the surface of the QDs. The bands of protonated amino groups on the surface of CdTe/CA QDs (NH_3_^+^) appear to overlap with a broad band corresponding to the OH stretching of water.

### 3.2. Photostability of CdTe/CA QDs

**Figure 3:**
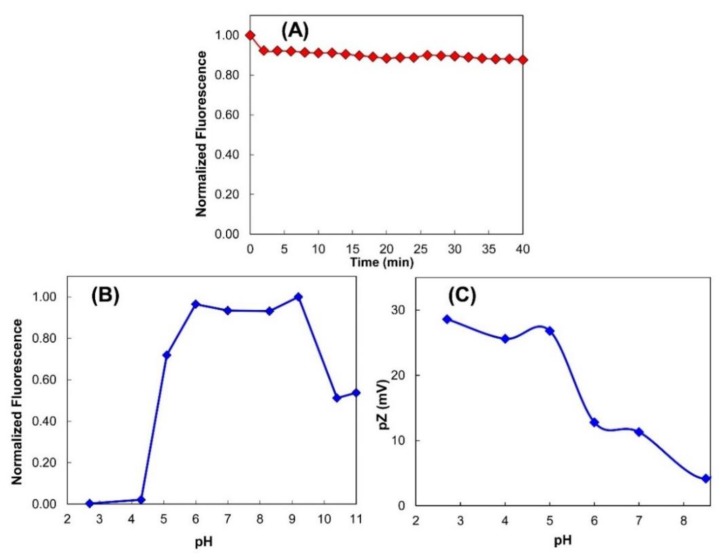
(**A**) Stability of CdTe/CA QDs, (**B**) normalized fluorescence of CdTe/CA QDs at different pH, and (**C**) variation of CdTe/CA QD zeta potential with pH.

The photostability of the CdTe/CA QDs was investigated by using the Time Driver system on a Horiba Jobin Yvon Nanolog spectrofluorometer (Figure 3A). As can be seen when the CA-coated CdTe nanoparticles were irradiated under a xenon ion laser for 40 min, the fluorescence intensity showed a decrease lower than 10%. The analyzed sample showed a good stability with no solid precipitated.

### 3.3. Effect of pH

The influence of pH on the fluorescent properties of the CdTe/CA QDs is shown in Figure 3B. As can be appreciated, the fluorescence behavior of the QDs is pH-dependent. The thiolated ligand that covers the QDs acts as a stabilizing agent of the nanocrystal, preventing aggregation and precipitation. In the acidic medium, the fluorescence intensity decreases as a result of the protonation of thiol groups and dissociation of the nanocrystal-ligand complexes on the surface of QDs. It has been reported that the ligands are removed from the surface of the QDs in the acidic medium [34,35]. As the pH increases, the thiol groups are deprotonated and the covalent bond between Cd and CA is strengthened, resulting in an increase in fluorescence intensity. At a higher pH (>9), the fluorescence intensity begins to decrease because the nanoparticles lose the positive charge and the repulsion processes between them decrease, leading to aggregation of QDs. In our study, fluorescence intensity remained practically constant between pH 6 and 9, so pH 8 (around physiological pH) was selected for further experiments.

The zeta potential of the CdTe QDs dispersed in water showed positive values throughout the pH range studied (Figure 3C), which confirms the presence of the positively charged amino groups on the surface of the QDs. The variation in the zeta potential with pH corresponds to the protonation/deprotonation of ethylamine groups (pKa = 10.63) on the surface of the nanoparticles [36].

### 3.4. Fluorescence Quenching of Thiol-Capped CdTe QDs by FA

**Figure 4:**
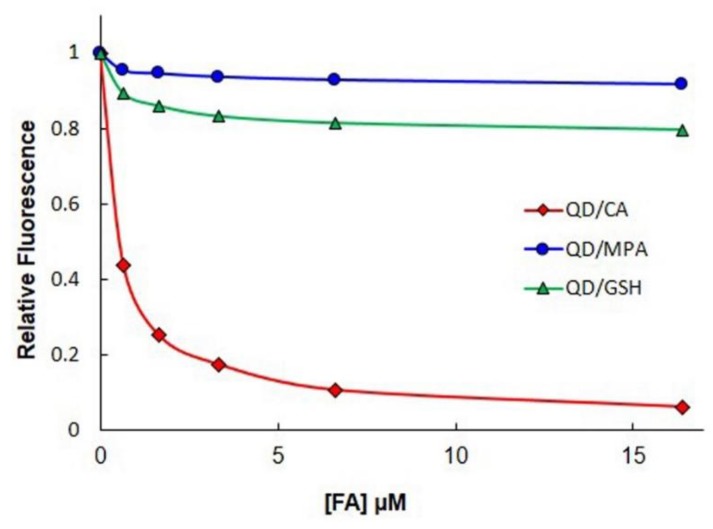
Response profile of CdTe QDs with different coating ligands (CA, mercaptopropionic acid (MPA), glutathione (GSH)) toward FA.

The behavior of the three thiol-capped CdTe QDs toward FA was studied. CdTe/GSH and CdTe/MPA QDs were selected of similar sizes as the CdTe/CA QDs studied (around 3.2 nm). QYs of CdTe/CA, CdTe/GSH, and CdTe/MPA QDs were determined to be 30, 41, and 49%, respectively. CdTe/GSH, CdTe/MPA, and CdTe/CA QDs were dispersed in PBS buffer solution at pH 8 and titrated by successive additions of FA. The fluorescence spectra were recorded in each addition and the response profile of each QD toward FA was obtained (Figure 4). The addition of FA to CdTe/MPA and CdTe/GSH QDs caused a fluorescence intensity quenching of only 10–15%. However, the quenching was noticeably greater in the QDs coated with CA, due to a greater attraction between the donor and the acceptor, favoring the possible electron transfer process. The theory of the photoinduced electron transfer is based on the assumption that the donor and acceptor orbitals overlap slightly at a separation distance of 7 Å [37]. The zeta potentials of CdTe/MPA, CdTe/GSH, and CdTe/CA QDs at pH 8 were −68, −14, and 7 mV respectively. The zeta potential values are due to the different structures of the thiolated ligands used as the QD coating. In all three cases, thiol groups bind strongly to the metal ions of QDs, leaving other groups on the surface of the nanoparticles. The MPA shows a carboxylic group with a pK_a1_ of 4.34 [38]. At pH 8, these carboxylic groups are deprotonated, so the particle is negatively charged. The GSH shows two carboxylic groups with pK_a1_ = 2.12 and pK_a2_ = 3.53, which are deprotonated at pH 8, but additionally has a protonated amine group with pK_a4_ = 9.62 [39]. The zeta potential of CdTe/GSH QDs is less negative than that of CdTe/MPA QDs, because the amine groups are protonated at pH 8. Finally, the CA shows amino groups, with a pK_a2_ = 10.75 [40], which are protonated at the working pH. This explains the positive zeta potential of the CdTe/CA QDs at pH 8. Figure S2 (Supplementary Material) shows the structures of the different thiolated ligands and their pKa values. 

**Figure 5:**
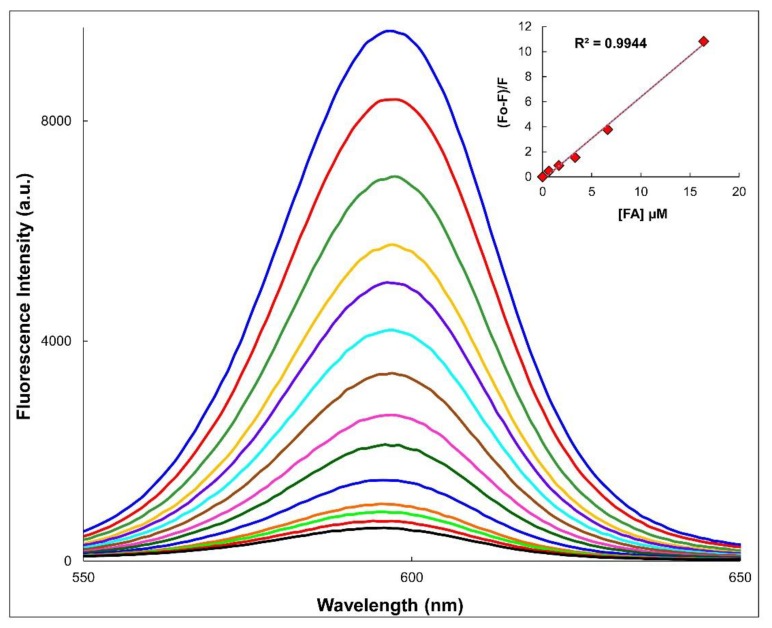
Fluorescence spectra of CdTe/CA QDs in PBS buffer solution (pH 8). Concentration range of FA = 0.16–16.4 µM. The inset shows the FA calibration curve (average from three repetitions).

The change in the photophysical properties of the fluorescent probe in the presence of FA was studied. Figure 5 shows the fluorescence spectra of CdTe/CA QDs during titration with FA in buffer solution at pH 8. As can be seen, the addition of FA in a concentration range between 0.16 and 33.3 μM caused a notable fluorescence quenching of the nanosensor (~95%), without an optical shift. 

The fluorescence of the CdTe/CA QDs varied linearly in the presence of FA in a concentration range from 0.16 to 16.4 µM. The inset shows the linear regression curve of FA detection (average from three repetitions). The detection limit of the method was 0.048 µM according to the equation of LOD = 3σ/s, where σ is the standard deviation of the blank (n = 11) and s is the slope of the calibration graph. The relative standard deviation (RSD) was 1.2% for the determination of 2 µM FA (n = 11). 

The Stern–Volmer equation F_o_/F = 1 + K_SV_ [FA] was used to describe fluorescence quenching. In this equation, F_o_ and F are the fluorescence intensities observed in the absence and presence of FA (quencher), respectively, and K_SV_ is the Stern–Volmer constant [41]. The relative fluorescence intensity, F_o_/F, exhibits downward curvature (Figure S3). A linear relationship between F_o_/F and the concentration of FA is observed when the concentration of FA is below 20 µM with R^2^ = 0.9944. The Stern–Volmer constant (K_SV_) calculated from the slope of the curve is 6.62 × 10^5^ M^−1^.

### 3.5. Mechanism of Quenching

The fluorescence of CdTe/CA QDs is described in terms of the semiconductor band theory [42]. According to this theory, an electron in the valence band (VB) is excited to the conduction band (CB) of the QDs, generating a “hole” of opposite charge to the excited electron. This pair is attracted to each other and is known as an “exciton.” When the excited electron returns to the valence band, recombination occurs, and fluorescence emission is generated. Therefore, changes on the surface of the QDs, such as the interaction with molecules and ions, can affect the efficiency of the electron–hole recombination process and produce a decrease in the fluorescence intensity of the QDs. In the case of FA, the observed pK_1_ = 2.38, pK_2_ = 3.38, and pK_3_ = 4.83 suggest the following dissociation sequence: N^1^H^+^, α-COOH, and γ-COOH, respectively [43]. As already indicated previously, at pH 8, QDs are positively charged due to the presence of the amino groups of CA on their surface, and the FA is negatively charged. As a result, the CA-capped QDs and the anionic FA form a stable assembly, with a binding constant of K = 5.2 × 10^5^ M^−1^ (Supplementary Information). As electronic transfer depends on the distance between the donor and acceptor, another aspect to consider is the length of the aliphatic chain attached to the amino group. A larger chain could cause a decrease in the efficiency of fluorescence quenching. In this process, not only do the opposite charges of the donor and acceptor influence, but so does the length of the aliphatic chain that acts as a spacer between FA and the surface of the QDs. 

To corroborate the interaction between the FA and CdTe/CA QDs, the zeta potential of the CdTe/CA QDs was measured upon addition of FA in the concentration range from 0 to 66 µM FA. As expected, the zeta potential became increasingly negative (from 7 to −12 mV), until there was no variation, indicating the interaction with protonated amine groups of CA on the surface of the nanoparticles. 

Different mechanisms have been proposed to explain fluorescence quenching of the QDs [44]. Among these are inner filter effects, energy transfer, and photoinduced electron transfer. The possibility of energy transfer in our work was ruled out because there was no overlap between the fluorescence emission spectrum of the QDs (Figure 2B) and the absorption spectrum of FA (Figure S4). We also exclude the internal filter mechanism, as the UV-visible spectrum of FA (Figure S4) does not show absorption at the excitation and emission wavelengths of the QDs. From this analysis, it is confirmed that the quenching is not due to the reabsorption processes. Then, the most likely reason for fluorescence quenching of QDs is photoinduced electron transfer from QDs to the FA, favored by the electrostatic association between the involved species, preventing the electron–hole recombination. Scheme 1 shows a schematic illustration of the quenching mechanism of FA detection.

#### Calculation of Free Energy (ΔGet) for the Electron Transfer Process

The energy change for the photoinduced electron transfer processes can be estimated with the following equation [41]:

∆*G* = *E*_ox_ (D) − *E*_red_ (A) − Δ*G*_00_ + C_coulombic_, (3)

where *E*_ox_ is the oxidation potential of the donor CdTe/CA QDs, *E*_red_ is the reduction potential of the acceptor FA, Δ*G*_00_ is the energy of the S_o_-S_1_ transition of the fluorophore (QDs), and C is the coulombic attraction of the ion pair [45]. If a negative ∆Get value is obtained, this indicates that the process is thermodynamically possible. The electrochemical behavior of CdTe QDs and FA has been reported in the literature. The considerable agreement between different results indicates that the voltammetric properties of CdTe QDs are intrinsic and are not greatly affected by the experimental conditions or the nature of the capping agent [46]. It has been reported that the anodic peak corresponding to the oxidation of the core of CdTe QDs (D = 3.2 nm) at pH 7 in phosphate buffer was observed around 0.74 V (vs. Ag/AgCl) [47], and a typical cyclic voltammogram (CV) of FA at pH 7.1 in phosphate buffer shows a first cathodic peak at −0.57 V (vs. Ag/AgCl) [48]. The Δ*G*_00_ value for the CdTe QDs obtained from the crossing points of the normalized fluorescence excitation and the emission spectra was 2.16 eV, and the coulombic term was neglected because it can be ignored in polar solvents [49]. As the Δ*G* thus calculated is negative (−0.85 eV), the process in the present system is thermodynamically possible. In conclusion, the coordinated behavior of the strong electrostatic interaction between CdTe/CA and FA and the electronic transfer from QDs to FA explains the mechanism of quenching. 

### 3.6. Selectivity of CA-Capped QD Sensor for FA Detection

**Figure 6:**
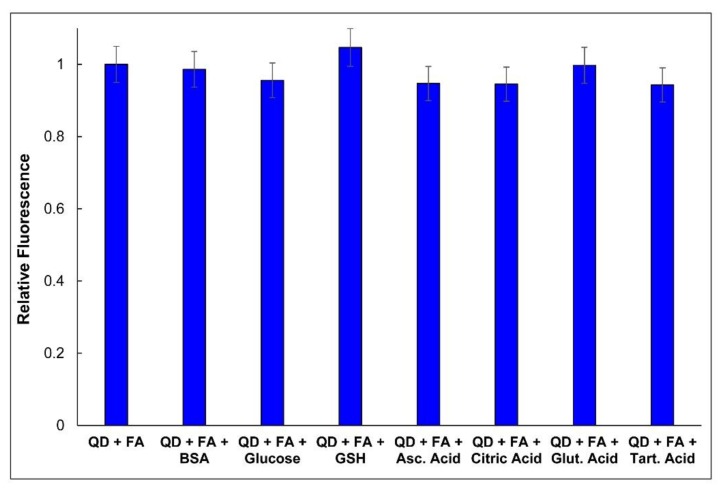
Results of the competition experiments in PBS buffer (pH = 8). Molar ratio of FA:Interference = 1:20.

The influence of possible interfering substances that have functional groups common to the analyte in their structures was examined. The study was carried out through competitive experiments in PBS buffer solution at pH 8. The fluorescence intensity of the CdTe/CA QDs solution was measured at 597 nm in the presence of FA (10 µM) and the corresponding interference (200 µM). The molar ratio of FA:Interference was 1:20. As can be appreciated in Figure 6, the relative fluorescence of QDs/CA (Y axis) showed a selective quenching effect by FA over other substances including bovine serum albumin (BSA), glucose, glutathione (GSH), ascorbic, citric, tartaric, and glutamic acids. This value is 1 for (QD + FA). In the presence of the corresponding interference QD + (FA + Interference), the relative fluorescence showed values close to 1, with an error of less than 5%. Therefore, the substances analyzed in ratio of FA:Interference = 1:20 do not represent an interference of the method.

The sensing mechanism is based on the electron transfer from the QDs to the FA. In the presence of an electron acceptor, exhibiting a LUMO energy level more positive than the conduction band potential, electron transfer quenching from the conduction band electrons to the acceptor units occurs. The selectivity of the recognition is due to the structure of the FA. In the first step, the electrostatic interaction between FA and CdTe/CA QDs places the acceptor and the donor at close range. The pteridine ring (Figure 1) has the N^3^H/CO fragment [43], which is positively charged and acts as an acceptor of electrons [50], favoring the electron transfer and the mechanism of fluorescence quenching. This electron-deficient group (pteridine ring) is not present in the other diacids studied.

Table 1 shows a comparison of different fluorescence methods for the detection of FA. As can be seen, the detection limit, RSD, and linearity of our probe are comparable or better than the methods reported for the determination of FA. It should be noted that the proposed sensor is sensitive enough for the determination of FA in other types of food samples, as well as from the biological and environmental area. In addition, our nanosensor is very easy to obtain and has advantages such as low cost and simple and rapid methodology.

### 3.7. Analysis of Real Samples with the Proposed Nanosensor

Orange drink samples of two different brands (sample 1 and sample 2) were selected to test the applicability of the nanosensor in the determination of the FA in real samples. The proposed system was applied to detect FA, using the standard addition method. FA was determined directly without previous treatment of the samples. The results of the sample analysis are shown in Table 2. The recovery tests were conducted by adding four different amounts of FA to each sample. The results are the mean of three parallel analyses. Recoveries varied from 98% to 104% with a relative standard deviation (RSD%) lower than 4.1, indicating the reliability of this CD/CA fluorescent probe for detection of FA in orange beverages. 

## 4. Conclusions

A new FA nanosensor based on amino-modified CdTe/CA QDs was successfully developed. Upon the interaction of this probe with FA, a notable fluorescence quenching was produced. The nanosensor exhibits sensitivity and selectivity, and is easily synthetized. This novel nanoprobe has the advantages of simplicity, rapidity, and low cost. The method was applied to the determination of FA in orange beverages with excellent results. A quenching mechanism was proposed, based on the coordinated behavior of a strong electrostatic interaction between CdTe/CA and FA and electronic transfer from the QDs to FA. A high efficiency of the electron transfer process was obtained by the interactions between the amino protonated groups at the QD surface and the carboxylate groups of FA at pH = 8. The method has potential to be applied in other types of samples. 

## Figures and Tables

**Table 1:** Comparison of different fluorescence methods for FA determination.

Sensor	Linear Range (µM)	Detection Limit (µM)	RSD %	Ref.
ANTS-anchored Zn-Al-CO_3_-LDH particles	1–200	0.1	2.0 (n = 5)	[8]
Au NCs /BSA	0.0075–75	0.041	1.49 (n = 5)	[23]
CdTe/TGA QDs	5–100	0.095	2.7 (n = 6)	[25]
ZnS:Cu/ZnS/MPA QDs	0–100	11	*	[26]
Formation of pterine-6-carboxilic acid	0.226–0.566	0.0045	4.6 (n = 11)	[51]
Tb^3+^-1,10 phenanthroline	0.022–0.226	0.007	1.2 (n = 7)	[52]
CuInS_2_ QDs	0.2–100	0.08	3.7 (n = 9)	[53]
PVA-Trp-CdTe QDs	0.09–1.19 pM	1.3 pM	*	[54]
AuNCs/BSA-AuNCs/Cys	0.11–2.27	0.065	*	[55]
CdTe/TGA QDs	9–272	0.19	1.59 (n = 6)	[56]
CdTe/CA QDs	0.16–16.4	0.048	1.2 (n = 11)	Present work

* Not reported.

**Table 2:** Determination results of FA in orange drink samples (n = 3).

Sample	Found (µM)	Added (µM)	Total Found (µM)	Recovery (%)	RSD % (n = 3)
**Sample 1**	0.88	0.66	1.56	101.32	2.7
		1.66	2.64	103.82	2.8
		3.32	4.13	98.28	2.5
		6.62	7.51	100.13	3.4
**Sample 2**	2.14	0.66	2.91	103.91	3.8
		1.66	3.86	101.54	2.9
		3.32	5.58	102.12	3.7
		6.62	8.61	98.32	4.1

## Supplementary Materials

The following are available online at https://www.mdpi.com/1424-8220/19/20/4548/s1, Figure S1: Evolution of absorption and fluorescence spectra of CdTe/GSH, CdTe/MPA, and CdTe/CA QDs at different reaction times; Figure S2: Structures and pK_a_ values of thiolated ligands (GSH, MPA, and CA); Figure S3: Stern–Volmer plot of the CdTe QDs-CA toward FA at 597 nm in PBS buffer (pH = 8); Figure S4: UV-visible absorption spectrum of FA; Table S1: Optical properties of CdTe QDs coated with MPA, GSH, and CA; Procedures: (a) Preparation and characterization of thiol-capped CdTe QDs; (b) determination of quantum yields (*Φ_x_*); (c) calculation of binding constant between QDs/CA and FA.
