# Supplementary material for: CdTe Quantum Dots Modified with Cysteamine: A New Efficient Nanosensor for the Determination of Folic Acid
Source: Sensors (Basel). 2019 Oct 19;19(20):4548. doi: 10.3390/s19204548 (PMC6832340; doi:10.3390/s19204548)
Supplement: Supplementary file 1 [file sensors-19-04548-s001.pdf]

sensors

Supplementary Material

CdTe Quantum Dots Modified with Cysteamine: A New Efficient Nanosensor for the Determination of Folic Acid

Doris E. Ramírez-Herrera [1], Ana Patricia Reyes-Cruzaley [1], Giselle Dominguez [2], Francisco Paraguay-Delgado [3], Antonio Tirado-Guízar [1] and Georgina Pina-Luis [1,*]

[1] Tecnológico Nacional de México/Instituto Tecnológico de Tijuana, Centro de Graduados e Investigación, A.P. 1166, Tijuana 22500, Mexico; doris_e_777@hotmail.com (D.E.R.-H.); ana.reyes@tectijuana.edu.mx (A.P.R.-C.); guizarantonio@gmail.com (A.T.-G.)

[2] Miami Dade College, North Campus, 11380 NW 27th Ave, Miami, FL 33167, USA; gdomingu@mdc.edu

[3] Centro de Investigación en Materiales Avanzados S. C., Departamento de Física de Materiales, Av. Miguel de Cervantes 120, Complejo Industrial Chihuahua, Chihuahua CP 31136, Mexico; francisco.paraguay@cimav.edu.mx

* Correspondence: gpinaluis@yahoo.com or gpinaluis@tectijuana.mx

Received: 13 September 2019; Accepted: 15 October 2019; Published: date

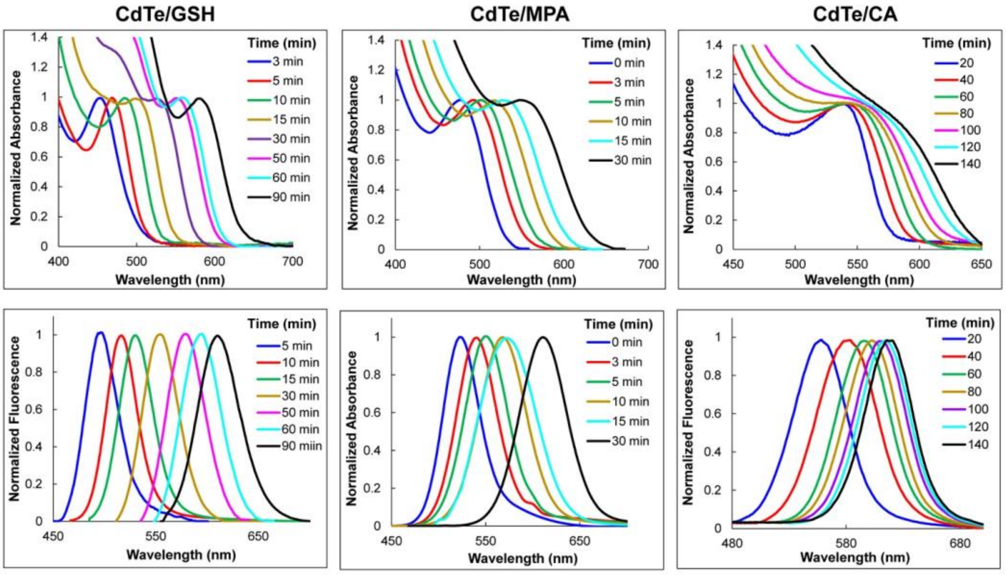

Figure S1. Evolution of absorption and fluorescence spectra of CdTe/GSH, CdTe/MPA and CdTe/CA QDs at different reaction times.

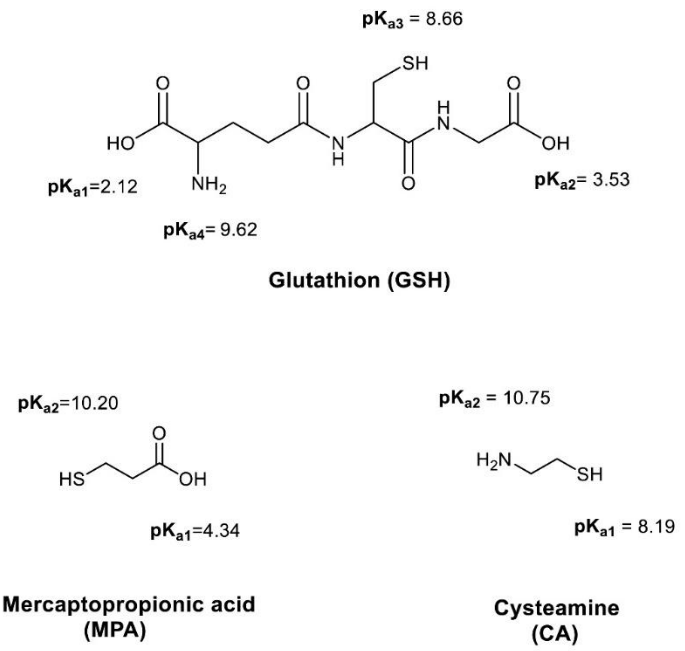

Figure S2. Structures and pKa values of thiolated ligands (GSH, MPA and CA).

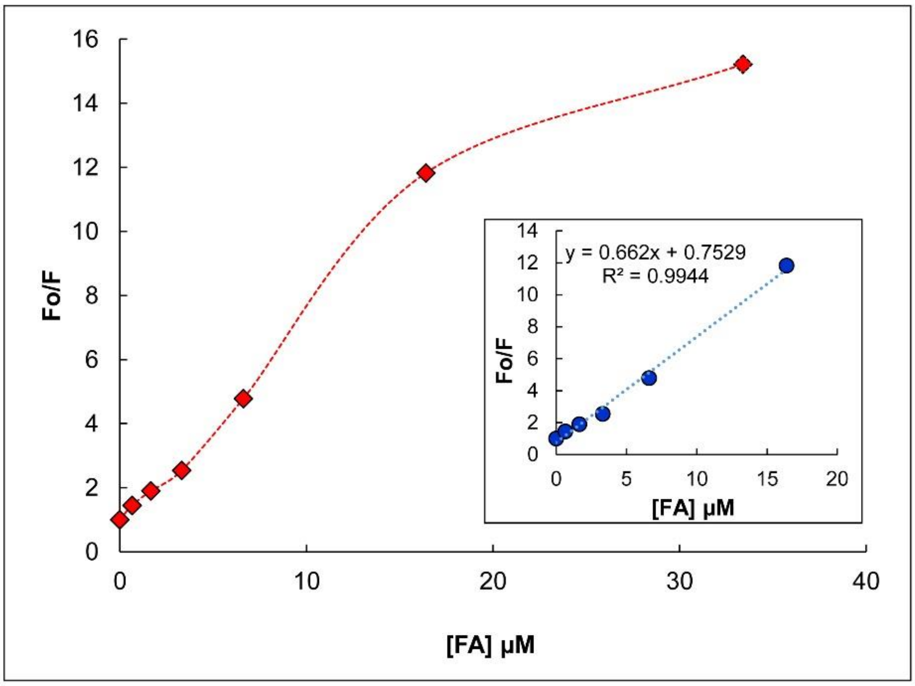

Figure S3. Stern-Volmer plot of the CdTe QDs-CA towards FA at 597 nm in PBS buffer (pH= 8).

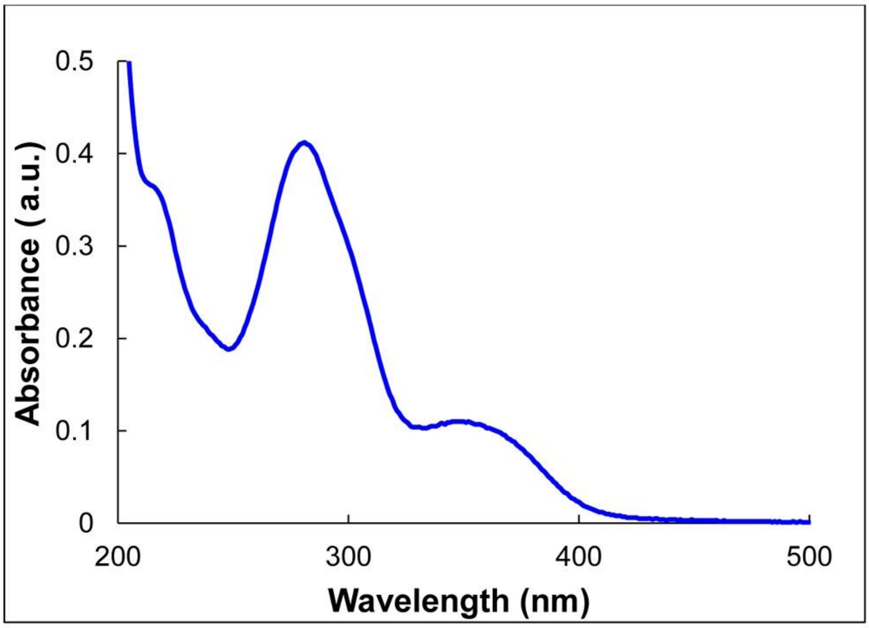

Figure S4. The UV-visible absorption spectrum of FA.

Table S1. Optical properties of CdTe QDs coated with MPA, GSH and CA.

Ligand	Reaction Time (min)	λ_{ABS} (nm)	Diameter (nm)*	Molar Absortivity ε ($Lmol^{-1}cm^{-1}$)	λ_{EM} (nm)
GSH	5	469	1.22	15414.6	498
	10	485	1.87	37902.7	516
	15	499	2.32	59573.2	530
	30	525	2.90	95877.8	555
	50	544	3.17	116216.9	581
	60	550	3.24	121562.7	595
	90	580	3.50	143371.7	610
MPA	3	477	1.57	26039.0.7	541
	5	493	2.13	49891.69	552
	10	502	2.40	64147.4	568
	15	527	2.93	98280.2	572
	30	547	3.21	118947.8	611
CA	20	539	3.11	111389.8	558
	40	542	3.15	114328.5	584
	60	545	3.19	117140.5	597
	80	550	3.24	121562.7	603
	100	555	3.29	125684.4	609
	120	558	3.32	128029.8	614
	140	564	3.38	132478.8	620

* Calculated using Peng regression [1]

Procedures

a) Preparation and characterization of thiol-capped CdTe QDs

QDs modified by GSH and MPA were synthetized according to a methodology used in previous works of the group [2]. Briefly, 0.4 mmol of thiolated ligand (GSH or MPA) and 0.4 mmol of $CdCl_2 \cdot 2.5H_2O$ were dissolved in 100 mL of deionized water in a three-necked flask. The mixture is subjected to magnetic stirring and the pH is adjusted to10 using 1M NaOH solution. After 5 min of vigorous stirring, a mixture formed by 0.08 mmol of K_2TeO_3 and 4.2 mmol of $NaBH_4$ in 100 mL of ionized water, is added into the three-necked flask in agitation. After another 5 min stirring, a condenser is attached to the flask and the mixture is refluxed at 100 °C. Aliquots were taken at different reaction times to obtain QDs of different sizes. Molar relation Cd/Te/LT/$NaBH_4$ was 1/0.2/1/10. The synthesis procedure of CdTe/CA QDs is similar, except that the pH value of Cd precursor solutions was adjusted to 5.6. The thiol-capped QDs solutions were concentrated and purified by ethanol precipitation and collected via centrifugation.

b) Determination of quantum yields (Φ_x)

The fluorescence quantum yields (QY) of the QDs were obtained by a methodology used in a previous article [2] by comparison with a fluorescence reference standard of fluorescein with a QY of 79% in 0.1 M NaOH using the equation:

$$\phi_{QD} = \phi_{FL}\left(\frac{Grad_{QD}}{Grad_{FL}}\right)\left(\frac{n_{QD}^2}{n_{FL}^2}\right) \quad (1)$$

where the subscripts *FL* and *QD* denote fluorescein and QD respectively, ϕ is the fluorescence quantum yield, *Grad* the gradient from the plot of integrated fluorescence intensity *vs* absorbance, and n the refractive index of the solvent.

QYs of CdTe/CA, CdTe/GSH and CdTe/MPA QDs of similar size (around 3.2 nm) were determined to be 30, 41 and 49 respectively.

c) Calculation of binding constant between QDs/CA and FA

The binding constant (K) between QD/CA and FA was determined using a modification of the Stern Volmer equation: [3]

$$\frac{1}{F_0 - F} = \frac{1}{F_0} + \frac{1}{KF_0[Q]}$$

where K is the binding constant of the electrostatic conjugate, Q is the concentration of FA, F_o and F are the fluorescence intensity in the absence and in the presence of FA respectively. The binding constant calculated was K = 5.2 x 10^5 M^{-1}

References

1. Yu, W.W.; Qu, L.H.; Guo, W.Z.; Peng, X.G. Experimental determination of the extinction coefficient of CdTe, CdSe and CdS nanocrystals. Chem Mater 2003, 15, 2854-2860.
2. Ramírez-Herrera, D. E.; Tirado-Guízar, A.; Paraguay-Delgado, F.; Pina-Luis, G. Ratiometric arginine assay based on FRET between CdTe quantum dots and Cresyl violet. *Microchim. Acta*, **2017**, *184*, 1997-2005.
3. Shen, X-C.; Liou, X-Y.; Ye, L-P.; Liang, H.; Wang, Z-Y. Spectroscopic studies on the interaction between human hemoglobin and CdS quantum dots. *J. Colloid Interf. Sci.* **2007**, *311*, 400-406.
